# Comparative Genomics of Bacteroides fragilis Group Isolates Reveals Species-Dependent Resistance Mechanisms and Validates Clinical Tools for Resistance Prediction

**DOI:** 10.1128/mbio.03603-21

**Published:** 2022-01-18

**Authors:** Miranda J. Wallace, Sophonie Jean, Meghan A. Wallace, Carey-Ann D. Burnham, Gautam Dantas

**Affiliations:** a Department of Pathology & Immunology, Division of Laboratory and Genomic Medicine, Washington University School of Medicine, St. Louis, MO, USA; b The Edison Family Center for Genome Sciences and Systems Biology, Washington University School of Medicine, St. Louis, MO, USA; c Department of Pathology and Laboratory Medicine, Nationwide Children’s Hospital, The Ohio State University Wexner Medical Center, Columbus, OH, USA; d Department of Medicine, Washington University School of Medicine, St. Louis, MO, USA; e Department of Pediatrics, Washington University School of Medicine, St. Louis, MO, USA; f Department of Molecular Microbiology, Washington University School of Medicine, St. Louis, MO, USA; g Department of Biomedical Engineering, Washington University in St. Louis, St. Louis, MO, USA; Northern Arizona University

**Keywords:** Bacteroides, anaerobes, antibiotic resistance, beta-lactams, carbapenems, genomics, mass spectrometry, taxonomy

## Abstract

Bacteroides fragilis group (BFG) are the most frequently recovered anaerobic bacteria from human infections, and resistance to frontline antibiotics is emerging. In the absence of routine antimicrobial susceptibility testing (AST) for BFG in most clinical settings, we assessed the utility of clinical and modern genomics tools to determine BFG species-level identification and resistance patterns. A total of 174 BFG clinical isolates supplemented with 20 archived carbapenem-resistant B. fragilis *sensu stricto* (BFSS) isolates underwent antimicrobial susceptibility testing, MALDI-ToF mass-spectrometry, and whole-genome sequencing (WGS). Bruker BioTyper and VITEK-MS MALDI-ToF systems demonstrated accurate species-level identifications (91% and 90% agreement, respectively) compared to average nucleotide identity (ANI) analysis of WGS data. Distinct β-lactamase gene profiles were observed between BFSS and non-*fragilis Bacteroides* species, with significantly higher MICs to piperacillin-tazobactam in B. vulgatus and B. thetaiotaomicron relative to BFSS (*P* ≤ 0.034). We also uncovered phylogenetic diversity at the genomospecies level between division I and division II BFSS (ANI <0.95) and demonstrate that division II BFSS strains harbor an increased capacity to achieve carbapenem resistance through chromosomal activation of the CfiA carbapenemase. Finally, we report that CfiA detection by the Bruker BioTyper Subtyping Module accurately detected carbapenem resistance in BFSS with positive and negative percent agreement of 94%/90% and 95%/95% compared to ertapenem and meropenem susceptibility, respectively. These comparative analyses indicate that resistance mechanisms are distinct at both the phenotypic and genomic level across species within the BFG and that modern MALDI-ToF identification systems can be used for accurate species-level identification and resistance prediction of the BFG.

## INTRODUCTION

The diagnosis and treatment of anaerobic infections pose several unique challenges. These infections are often polymicrobial and caused by an array of species that can be slow-growing or difficult to cultivate and require specialized specimen collection techniques (1). Bacteroides fragilis group (BFG) species are the most frequently isolated organisms from anaerobic infections and can be recovered from various sites, including abscesses and bloodstream infections (2). A common source of anaerobic infection is the commensal microbiota, of which *Bacteroides* and *Parabacteroides* are found in high abundance in the gastrointestinal tract of healthy humans (3).

Increases in the incidence of anaerobic infection have been observed in recent years (4), and antimicrobial resistance is associated with increased morbidity and mortality (5). Most clinical laboratories do not perform antimicrobial susceptibility testing for anaerobic bacteria. Infections with BFG are typically treated empirically with agents such as metronidazole, β-lactam/β-lactamase inhibitor combinations, or carbapenems (6). However, resistance to β-lactam/β-lactamase inhibitor combinations and carbapenems is emerging in BFG organisms, and resistance to metronidazole and other antibiotic classes have also been described (7–10). Antibiotic susceptibility surveys have suggested species-specific trends in phenotypic resistance among BFG organisms (8). Furthermore, B. fragilis *sensu stricto* (BFSS) is most associated with carbapenem resistance and can be categorized into two divisions based on the absence (division I) or presence (division II) of the chromosomally encoded carbapenemase gene, *cfiA* (11). Large-scale genomic studies are lacking for BFG bacteria, which could validate the accuracy of clinical tools routinely used to identify members of BFG to the species level. Furthermore, comparative resistome studies within a large and taxonomically diverse collection of BFG bacteria could inform distinct resistance mechanisms underlying previously observed species-dependent resistance profiles.

Here, we present a comprehensive clinical and comparative genomics study of a collection of 174 BFG isolates collected in St. Louis, MO from 2018 to 2020, supplemented with 20 carbapenem-resistant BFSS isolates. We performed species-level identification, phenotypic susceptibility testing, and whole-genome sequencing (WGS) on this collection of isolates. Matrix-assisted laser desorption ionization time of flight mass spectrometry (MALDI-ToF) species-level identifications were confirmed using comparative genomics. We report distinct phenotypic and genotypic resistance profiles among BFG species against frontline antibiotic agents, bolstering the value of routine species-level reporting of BFG in clinical practice. Finally, we analyzed annotated antibiotic resistance genes (ARGs) against relevant antibiotics in this isolate collection and expanded the characterization of the genetic distinction between division I and division II BFSS. Our data evaluated the reliability of several clinical tools to accurately determine both BFG species and resistance profiles while providing a comprehensive landscape of the antibiotic resistome in BFG with special attention toward commonly utilized anaerobic treatment options.

## RESULTS

### Comparison of WGS and MALDI-TOF MS characterization of BFG isolates.

**FIG 1:**
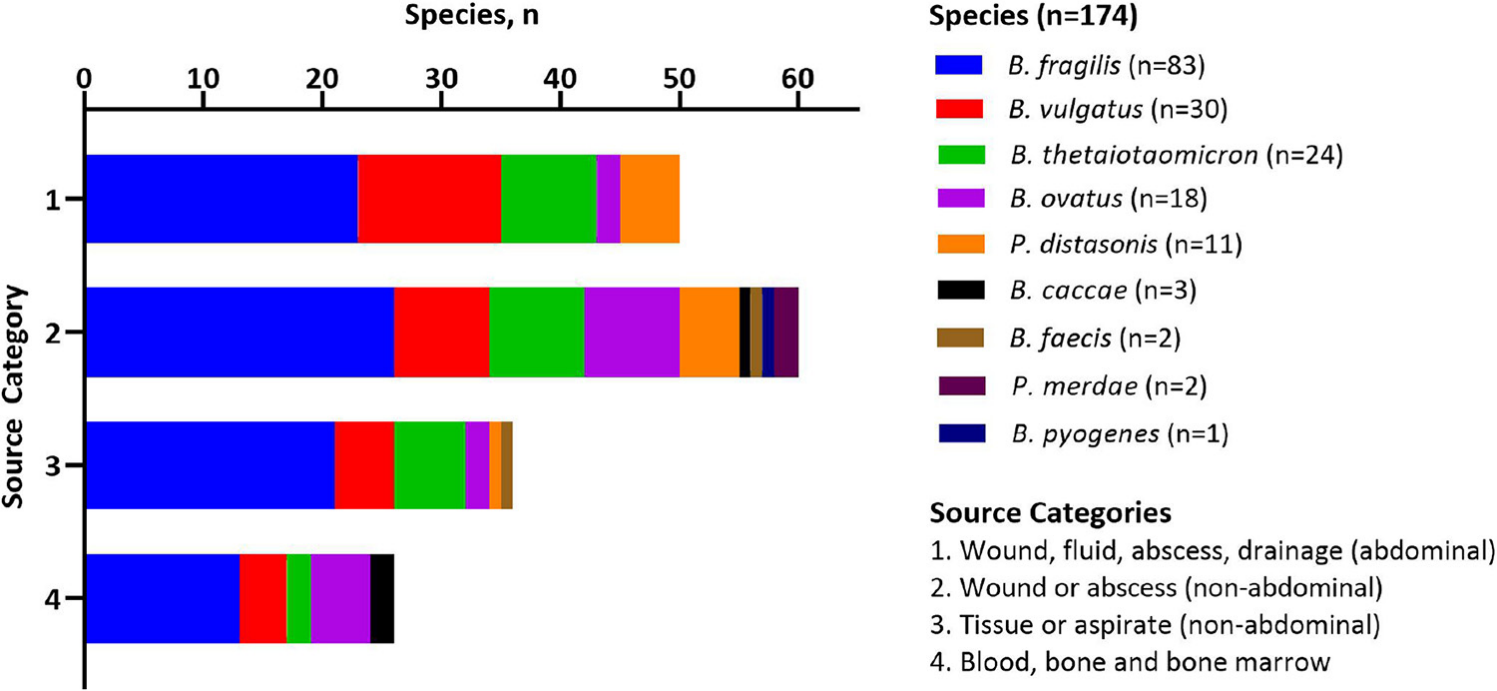
Infection sources and species distribution of 174 B. fragilis group (BFG) isolates collected from patient specimens submitted to the Barnes-Jewish Hospital in St. Louis, Missouri. Species data from Bruker Biotyper MALDI-ToF MS are shown.

A total of 174 BFG isolates recovered from patient samples submitted to the Barnes-Jewish Hospital in St. Louis, Missouri from 2018 to 2020 during routine clinical care were evaluated in this study (Fig. 1, see Materials and Methods). Isolates were recovered from a variety of sources that were grouped into 4 categories: 1, wound, fluid, abscess, or drainage from abdominal sites (*n* = 50); 2, wound or abscess from non-abdominal sites (*n* = 60); 3, tissue or aspirate from non-abdominal sites (*n* = 36); 4, blood, bone, and bone marrow (*n* = 26) (Data Set S1). MALDI-TOF MS analysis was performed on all isolates using the Bruker BioTyper (Billerica, MA) and bioMérieux VITEK-MS (Durham, NC) (Data Set S1). Among the isolates, 9 unique BFG species were isolated representing both *Bacteroides* and *Parabacteroides* genera (Fig. 1). Most isolates analyzed were from wounds and abscesses from both abdominal and non-abdominal sites (Fig. 1). The distribution of BFG species was similar between isolate source groupings with approximately half of the isolates identified as BFSS (*n* = 83), followed by B. vulgatus (*n* = 30), B. thetaiotaomicron (*n* = 24), B. ovatus (*n* = 18), and P. distasonis (*n* = 11).

10.1128/mbio.03603-21.7 DATA SET S1 Clinical metadata associated with Bacteroides fragilis group isolates in this study. Download Data Set S1, XLSX file, 0.02 MB. Copyright © 2022 Wallace et al. 2022 Wallace et al. https://creativecommons.org/licenses/by/4.0/ This content is distributed under the terms of the Creative Commons Attribution 4.0 International license.

**FIG 2:**
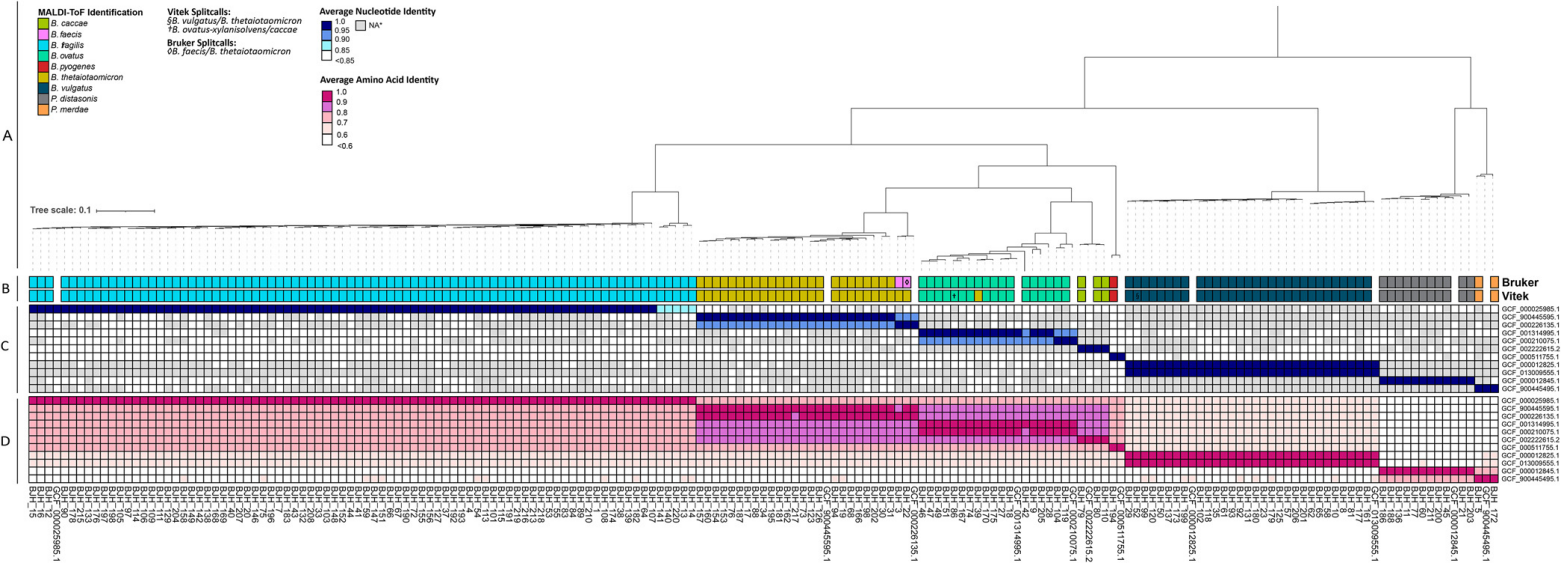
Taxonomic analysis of the BFG isolate collection from Barnes-Jewish Hospital. (A) Phylogenetic tree of complete BFG isolate collection generated from core genome alignment with type assemblies. (B) Species-level identifications determined by two commonly utilized MALDI-ToF technologies. (C) Pairwise average nucleotide identity (ANI) and pairwise average amino acid identity (D) values for the BJH isolates compared with type assemblies. *ANI values for isolate pairs with percentage identity >0.85 that did not have adequate genome coverage (coverage <0.5) following pyani analysis were excluded.

BFG bacteria are taxonomically diverse, consisting of at least 24 species from two distinct families (Bacteroidaceae and Tannerellaceae) (2). To confirm the accuracy of species-level identification of the Bruker BioTyper and VITEK-MS platforms, we performed Illumina short-read WGS of the entire isolate collection. *De novo* assemblies were generated from the WGS data (see Materials and Methods and Fig. S1) and used to perform phylogenomic analyses, which were compared directly with MALDI-ToF identifications (Fig. 2). On a phylogenetic tree generated from a core genome alignment of isolate and type BFG assemblies, each isolate clustered with type assemblies matching most of the Bruker isolate identifications and VITEK-MS identifications. Exceptions included five BFSS isolates forming a clade independent of the BFSS type assembly as well as two isolates exhibiting discordant species identifications between the two MALDI-ToF systems: BJH_3 (Bruker: B. faecis, VITEK-MS: B. thetaiotaomicron) and BJH_39 (Bruker: *B. ovatus*, VITEK-MS: B. thetaiotaomicron). BJH_3 formed a clade with a B. faecis type assembly and shared >95% average nucleotide identity (ANI) with this assembly. Furthermore, by the same metric BJH_39 appeared to be B. ovatus. B. ovatus, B. faecis, and B. thetaiotaomicron reside in adjacent clades in the phylogenetic tree (Fig. 2), which may explain the discordant identifications within these species (12). Further exceptions were represented by two strains identified as B. ovatus by both Bruker and VITEK but shared 98% ANI with a B. xylanisolvens type assembly in contrast to only sharing 94% ANI with a B. ovatus type assembly (Fig. 2). Additionally, eight isolates originally identified as B. vulgatus had a higher pairwise ANI with a B. dorei type assembly and formed a distinct clade. However, the ANI threshold for the species-level identity of each of these eight isolates was met with each strain respective to type assemblies from both B. vulgatus and B. dorei species (ANI >95%) (Fig. S2) (12). Altogether, the percentage agreement between MALDI-ToF species callout and species determination via ANI was 91% (159/174) for Bruker and 90% (156/174) for VITEK-MS. The five BFSS isolates forming a distinct clade exhibited ANI within the range of 0.85 to 0.90 relative to the BFSS type assembly GCF_000025985. All isolates had >60% average amino acid identity (AAI) with the respective type assemblies, the meeting proposed genus-level AAI cutoffs (13, 14) within *Bacteroides* or *Parabacteroides*.

10.1128/mbio.03603-21.1 FIG S1 Genome assembly metrics, including (A) number of contigs, (B) N50, (C) average coverage, (D) completeness/contamination determined with checkM (31). Download FIG S1, TIF file, 2.1 MB. Copyright © 2022 Wallace et al. 2022 Wallace et al. https://creativecommons.org/licenses/by/4.0/ This content is distributed under the terms of the Creative Commons Attribution 4.0 International license.

10.1128/mbio.03603-21.2 FIG S2 Pairwise ANI values for isolates identified as Bacteroides vulgatus by Bruker and VITEK MALDI-ToF technology. Clades from Fig. 2 that contain type strains for Bacteroides vulgatus (GCF_000012825.1, [A]) and Bacteroides dorei (GCF_013009555.1, [B]) are shown. Download FIG S2, TIF file, 0.4 MB. Copyright © 2022 Wallace et al. 2022 Wallace et al. https://creativecommons.org/licenses/by/4.0/ This content is distributed under the terms of the Creative Commons Attribution 4.0 International license.

### Antimicrobial resistance profiles are distinct across BFG species.

**FIG 3:**
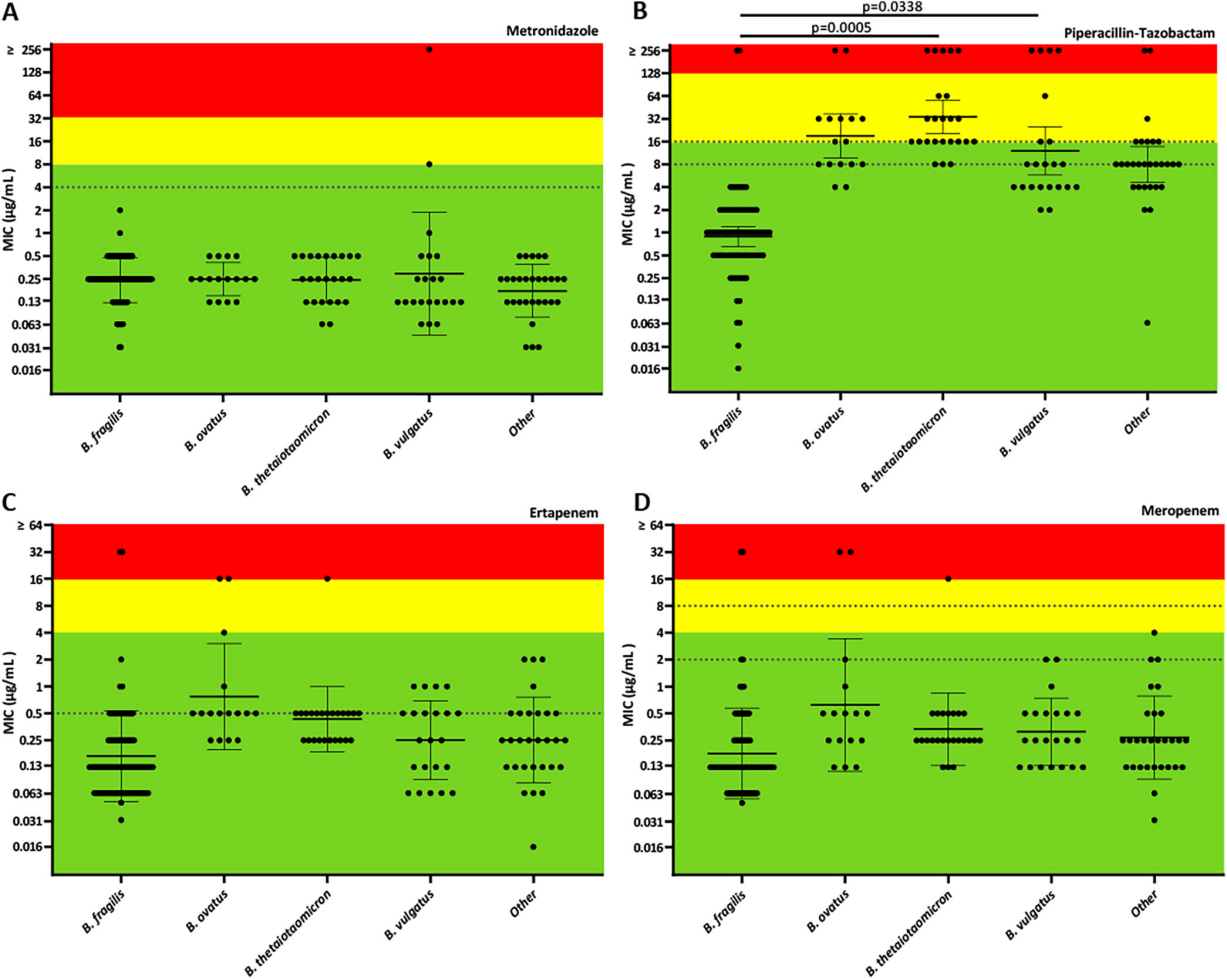
Scatterplot of MICs of BJH isolates to select antibiotic agents: (A) Metronidazole. (B) Piperacillin-Tazobactam. (C) Ertapenem. (D) Meropenem. Green, yellow, and red regions represent susceptible, intermediate, and resistant interpretations, respectively, according to CLSI guidelines (34). Dashed lines represent EUCAST clinical breakpoints (35). Geometric mean and 95% confidence interval are plotted with statistically significant adjusted *P* values (*P* < 0.05) shown. Statistical analysis was performed using ordinary one-way ANOVA with Tukey’s multiple-comparison test to compare the means of each species group using Prism v9.0.0. Species groupings for the isolates are based on average nucleotide identities relative to type genomes.

Susceptibility testing was performed using gradient diffusion for four commonly prescribed anaerobic agents, including metronidazole, piperacillin-tazobactam, meropenem, and ertapenem (Fig. 3 and Table 1). Carbapenem resistance was detected but uncommon in our collection; however, increased carbapenem resistance among BFSS has been reported (7). Our clinical isolates were supplemented with a collection of BFSS strains enriched for carbapenem resistance from the IHMA strain bank (Data Set S1). Statistical differences in the mean MIC between all species groups were calculated using ordinary one-way ANOVA with Tukey’s multiple-comparison test to compare each species group. Geometric mean metronidazole MIC values did not significantly differ between all species (*P* ≥0.2207) (Fig. 3A). However, MICs to β-lactam antibiotics were uniformly elevated in non-*fragilis Bacteroides* species relative to BFSS (Fig. 3B to D). The piperacillin-tazobactam MICs were significantly elevated for B. thetaiotaomicron isolates (*P* = 0.005; MIC_50_ = 16, MIC_90_ = 256 μg/mL), followed by B. ovatus (MIC_50_ = 16, MIC_90_ = 32 μg/mL) and B. vulgatus (MIC_50_ = 8, MIC_90_ = 256 μg/mL) (*P* = 0.0338), suggesting that β-lactam resistance is more common among non-*fragilis* species. Non-susceptibility to any β-lactam agent tested was rare among BFSS BJH isolates, and, as expected, IHMA isolates were mostly ertapenem (MIC_50_ = 16, MIC_90_ > 32 μg/mL), meropenem (MIC_50/90_ > 32 μg/mL), and piperacillin-tazobactam resistant (MIC_50_ = 64, MIC_90_ > 256 μg/mL) (Table 1).

**TABLE 1:** Antimicrobial susceptibility data for Bacteroides fragilis group isolates collected at the Barnes-Jewish Hospital in St. Louis, MO

	Piperacillin/tazobactam CLSI^*a*^: S, MIC ≤16 μg/mL EUCAST: S, MIC ≤8 μg/mL				Ertapenem CLSI: S, MIC ≤4 μg/mL EUCAST: S, MIC ≤0.5 μg/mL				Meropenem CLSI: S, MIC ≤4 μg/mL EUCAST: S, MIC ≤2 μg/mL				Metronidazole CLSI: S, MIC ≤8 μg/mL EUCAST: S, MIC ≤4 μg/mL			
	MIC50	MIC90	CLSI-%S	EU-%S	MIC50	MIC90	CLSI-%S	EU-%S	MIC50	MIC90	CLSI-%S	EU-%S	MIC50	MIC90	CLSI-%S	EU-%S
B. fragilis (*n* = 83)	1	4	98	98	0.125	0.5	98	94	0.125	0.5	98	98	0.25	0.5	100	100
B. vulgatus (*n* = 22)	8	256	77	68	0.25	1	100	82	0.25	1	100	100	0.125	1	95	91
B. thetaiotaomicron (*n* = 24)	16	256	50	13	0.5	0.5	96	96	0.25	0.5	96	96	0.25	0.5	100	100
B. ovatus (*n* = 16)	16	256	56	44	0.5	16	88	75	0.5	32	88	88	0.25	0.5	100	100
Parabacteroides distasonis (*n* = 11)	0.25	0.5	100	100	0.25	1	100	82	0.125	1	100	100	0.25	0.5	100	100
Other Bacteroides fragilis group^*b*^ (*n* = 18)	8	256	83	61	0.25	2	100	89	0.25	2	100	100	0.125	0.25	100	100
B. caccae (*n* = 3)	8	8	100	100	0.25	0.25	100	100	0.125	0.125	100	100	0.25	0.25	100	100
B. faecis (*n* = 2)	32	256	0	0	0.5	2	100	50	0.25	1	100	100	0.032	0.25	100	100
B. pyogenes (*n* = 1)	NA	N/A	100	100	NA	N/A	100	100	NA	N/A	100	100	NA	N/A	100	100
P. merdae (*n* = 2)	4	4	100	100	0.25	0.5	100	100	0.25	0.5	100	100	0.25	0.25	100	100

a Abbreviations: CLSI, Clinical and Laboratory Standards Institute; EUCAST, European Union Committee on Antimicrobial Susceptibility Testing; S, susceptible.

b B. dorei (*n* = 8), B. caccae (*n* = 3), B. faecis (*n* = 2), B. xylanisolvens (*n* = 2), B. pyogenes (*n* = 1), P. merdae (*n* = 2).

**FIG 4:**
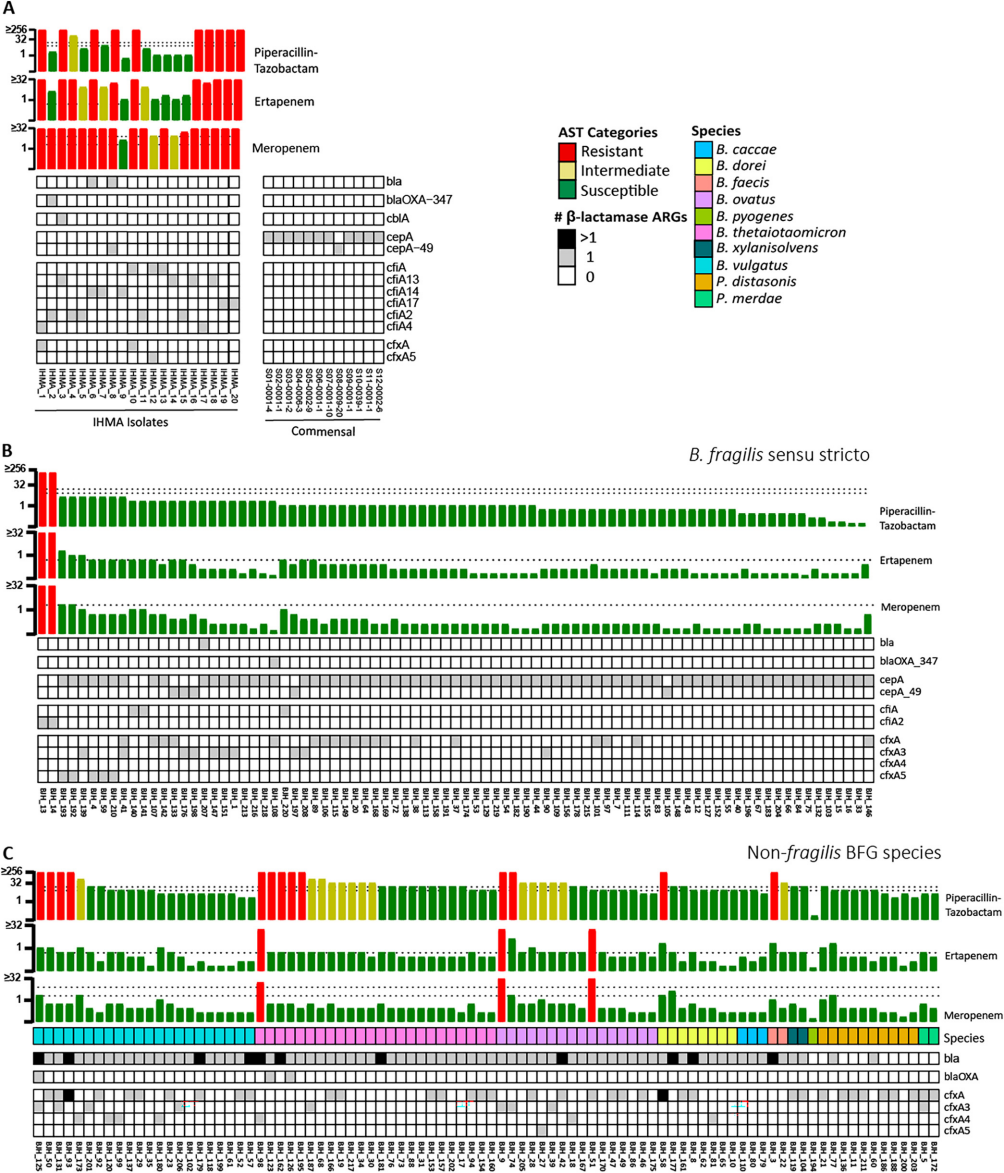
Phenotypic and genotypic resistance to β-lactam classes for (A) BFSS IHMA isolates and representative commensal BFSS genomes (16), (B) BFSS isolates collected at Barnes-Jewish Hospital (BJH) in St. Louis, MO, and (C) non-BFSS BFG isolates also collected at BJH. Isolates are ordered by their phenotypic resistance profile to piperacillin-tazobactam; AST bars are colored based on the CLSI interpretations (34) with dashed lines representing EUCAST breakpoints (35).

To identify potential mechanisms of resistance, AMRfinder (15) was employed to interrogate the ARG profile of these strains (Fig. 4 and Fig. S3). A collection of previously reported commensal BFSS assemblies from 12 individuals was also included to understand the baseline ARG profile of this species (16). All 205 BFG genomes encoded a rich population of ARGs against multiple classes, including tetracyclines, macrolides, and β-lactams (Fig. S3A). Distinct β-lactamase distribution patterns were observed among BFSS, non-*fragilis Bacteroides* species, and *Parabacteroides* species (Fig. 4). BFSS isolates exclusively harbored either the *cepA* or *cfiA* gene, which indicates the categorization of each strain as division I (*cepA*+/*cfiA*-) or division II (*cepA*-/*cfiA*+) (Fig. 4A and B) (11). The representative commensal BFSS genomes only encoded the *cepA* β-lactamase gene. In contrast, clinical non-*fragilis Bacteroides* isolates were enriched for class A β-lactamase genes identified through the Hidden Markov Model (HMM) profiling function of AMRfinder (*bla* genes, Fig. 4C). These *bla* genes shared ≥27.3% amino acid identity and formed approximate species-specific clusters, with a closely related multispecies clade also observed (Fig. S4). Cefoxitin resistance genes, *cfx*, were abundant in major non-*fragilis* BFG species (B. vulgatus, B. thetaiotaomicron, and B. ovatus), and were enriched in BFSS with elevated β-lactam resistance. For BFSS, phenotypic carbapenem resistance was only observed in the presence of the *cfiA* gene, except for IHMA_8 which had both *cepA* and an HMM-identified *bla* gene conserved among multiple species (Fig. 4A and B, Fig. S4A). Among all BFG isolates, a higher total number of β-lactamases was often associated with increased MICs to piperacillin-tazobactam (Fig. 4), especially in the case of *cfx*-positive BFSS strains (Fig. 4A and B).

10.1128/mbio.03603-21.3 FIG S3 Antimicrobial resistance elements in BFG isolates. (A) Complete presence/absence heatmap of resistance genes detected by AMRfinder v3.8.4. Presence/absence heatmap of known PBPs (B) and porins (C) in BFSS detected by BLASTP. The percentage identity of each PBP (D) and porin (E) relative to the following query sequences: CAH09986.1 (DD-DPasa) (26); CAH07970.1 (PBP1abBfr) (26); CAH07065.1 (PBP1cBfr) (26); CAH06034.1 (PBP2Bfr) (26); CAH06251.1 (PBP2xBfr) (26); CAH09590.1 (PBP3Bfr) (26); CAH05803.1 (PBP4Bfr) (26); CAH09813.1 (PBP7Bfr) (26); WP_032587333.1 (Bfr_porin_1); OCR43985.1 (Bfr_porin_2); OCR39813.1 (Bfr_porin_3); OCR39182.1 (Bfr_porin_4); AAK38604.1 (Omp121) (36); WP_005786951.1 (Omp71) (36); WP_005791792.1 (OmpA1) (37); WP_005786002.1 (OmpA3) (37); WP_005786560.1 (OmpA4_1) (37); WP_032575208.1 (OmpA4_2) (37). Download FIG S3, TIF file, 2.8 MB. Copyright © 2022 Wallace et al. 2022 Wallace et al. https://creativecommons.org/licenses/by/4.0/ This content is distributed under the terms of the Creative Commons Attribution 4.0 International license.

10.1128/mbio.03603-21.4 FIG S4 Cryptic *bla* genes identified in BFG isolates. (A) Neighbor-joining tree generated from MUSCLE alignment of amino acid sequences derived from *bla* genes identified through the Hidden-Markov Modeling function of AMRfinder (15). Multispecies clade is boxed. (B) MUSCLE alignment of consensus sequences generated from the multispecies clade, B. vulgatus, B. thetaiotaomicron, and *B. ovatus* with CepA and PER-1 class A β-lactamases. Amino acid motifs characteristic of class A β-lactamases (38) are boxed in red. Download FIG S4, TIF file, 1.8 MB. Copyright © 2022 Wallace et al. 2022 Wallace et al. https://creativecommons.org/licenses/by/4.0/ This content is distributed under the terms of the Creative Commons Attribution 4.0 International license.

### Insertion sequences activate cfiA-mediated carbapenem resistance in B. fragilis SS.

The presence of the *cfiA* gene alone does not typically correspond to high-level carbapenem resistance in BFSS due to inefficient gene expression from the native promoter (17). However, insertion sequences (IS) upstream of the gene can increase *cfiA* expression and result in high-level carbapenem resistance (10, 17). To identify the mechanism of carbapenem resistance in this cohort and validate available tools to determine phenotypic resistance, we compared genomic *cfiA* and upstream IS identifications with the Bruker MALDI Biotyper Subtyping module (MBT-S) and previously reported PCR-based screening methods that detect both *cfiA* and associated ISs (18).

Six percent (5/83) of BFSS isolates from BJH and 95% (19/20) of IHMA isolates had *cfiA* detected via AMRfinder in the WGS assemblies (Fig. 4); however, the respective contigs were often immediately interrupted upstream of *cfiA*. IS elements are known to interrupt short-read assemblies due to the presence of redundant IS copies throughout the genome (17). Thus, Oxford Nanopore long-read sequencing reads were generated to build hybrid assemblies for all *cfiA*-positive BFSS strains with contigs interrupted immediately upstream of *cfiA* (17/24). IS elements upstream of *cfiA* within the hybrid assemblies were identified with Prokka (19) which utilizes the ISfinder database (20). IS elements were identified upstream of *cfiA* in 12 BFSS genomic assemblies (Table 2), all of which were carbapenem-resistant according to both CLSI and EUCAST breakpoints. Most of the identified insertion sequences were in the IS1380 family (21). The *cfiA*-positive, carbapenem-resistant strain IHMA_10 had no associated IS as detected by ISfinder. However, a nucleotide BLAST search of the upstream region identified a transposase IS982 from Barnesiella viscericola, an obligate anaerobic Gram-negative bacterium related to Parabacteroides distasonis (22), suggesting cross-family transfer of IS elements.

**TABLE 2:** CfiA carbapenemase and associated insertion sequence detection in B. fragilis *sensu stricto* isolates with phenotypic or genotypic carbapenem resistance determinants

Strain	72 h MIC (μg/mL)		*cfiA* detection			IS detection				
	Ertapenem	Meropenem	MALDI	PCR	AMRFinder	PCR	ISfinder	Family	Identities	Origin
BJH_13	>32	>32	+	+	*cfiA2*	+	IS612B	IS1380	1595/1596	Bacteroides fragilis
BJH_14	>32	>32	+	+	*cfiA2*	+	IS612B	IS1380	1595/1596	Bacteroides fragilis
BJH_140	0.5	1	+	+	*cfiA*	−	Partial ISBf9	−	−	−
BJH_141	0.5	1	+	+	*cfiA*	−	Partial ISBf9	−	−	−
BJH_220	0.5	1	+	+	*cfiA*	−	−	−	−	−
IHMA_1	>32	>32	+	+	*cfiA4*	+	IS613	IS1380	1595/1595	Bacteroides fragilis
IHMA_2	4	>32	+	+	*cfiA2*	−	None detected			
IHMA_3	>32	>32	+	+	*cfiA13*	+	IS942	IS1380	1592/1598	Bacteroides fragilis
IHMA_4	32	>32	+	+	*cfiA2*	+	IS1169	IS5	1301/1317	Bacteroides fragilis
IHMA_5	8	32	+	+	*cfiA2*	−	−	−	−	−
IHMA_6	>32	>32	+	+	*cfiA14*	+	IS616	IS1380	1691/1691	Bacteroides fragilis
IHMA_7	8	>32	+	+	*cfiA14*	−	−	−	−	*−*
IHMA_8	16	>32	−	−	−	−	−	−	−	*−*
IHMA_9	1	4	+	+	*cfiA14*	−	Partial ISBf9	−	−	−
IHMA_10	>32	>32	+	+	*cfiA*	+	IS982^*#*^	IS982	−	Barnesiella viscericola
IHMA_11	8	>32	+	+	*cfiA2*	−	−	−	−	*−*
IHMA_12	1	8	+	+	*cfiA*	−	−	−	−	*−*
IHMA_13	2	32	+	+	*cfiA*	−	−	−	−	−
IHMA_14	1	8	+	+	*cfiA13*	−	−	−	−	−
IHMA_15	2	16	+	+	*cfiA2*	−	−	−	−	−
IHMA_16	>32	>32	+	+	*cfiA13*	−	IS614B	IS1380	1532/1597	Bacteroides fragilis
IHMA_17	16	>32	+	+	*cfiA4*	+	IS4351	IS30	1155/1155	Bacteroides fragilis
IHMA_18	>32	>32	+	+	*cfiA13*	+	IS614	IS1380	1585/1596	Bacteroides fragilis
IHMA_19	>32	>32	+	+	*cfiA17*	+	ISBf11	IS1380	1457/1594	Bacteroides fragilis
IHMA_20	>32	>32	+	+	*cfiA17*	+	ISBf11	IS1380	1457/1594	Bacteroides fragilis

NT = Not tested.

# Reverse-facing IS982 transposase identified using BLAST search of region upstream of *cfiA*, 99% identity with NCBI accession number WP_025278340.

The Bruker MALDI Biotyper Subtyping Module (MBT-S) is a novel MALDI-ToF application that enables rapid detection of specific bacterial resistance markers, including CfiA, at the same time during organism identification in a streamlined workflow. Following high-confidence identification of BFSS (i.e., a MALDI-TOF score ≥ 2.0), the subtyping module looks for the presence of peaks associated with expression of the CfiA carbapenemase and may, thus, serve as an early warning system of carbapenem-resistant BFSS isolates in clinical settings. We evaluated the accuracy of the MBT-S CfiA call-out with all BFSS isolates from both BJH and IHMA collections (*n* = 103) compared to phenotypic resistance evaluated using CLSI and EUCAST breakpoints (Table 3 and Table S1). Compared to ertapenem and meropenem resistance defined by CLSI breakpoints, the MBT-S demonstrated positive percent agreement (PPA), negative percent agreement (NPA) of 94%, 90%, and 95%, 95%, respectively. Using EUCAST interpretive criteria, the MBT-S demonstrated PPA, NPA of 84%, 96% for ertapenem, and 95%, 96% for meropenem, respectively. Among consecutively collected BFSS isolates from BJH only, the positive predictive value (PPV) was 50% and negative predictive values (NPV) ranged from 96 to 100% across all carbapenems and guidelines evaluated indicating the need for confirmatory testing of MBT-S CfiA positive callouts but high-confidence in MBT-S CfiA-negative callouts (Table S1). In settings of high rates of carbapenem resistance, PPV is expected to improve (Table S1). Category agreement (CA), very major error (VME), and major error (ME) rates were also determined for the MBT-S CfiA callout compared to carbapenem susceptibility interpreted using CLSI and EUCAST breakpoints. CA was defined as the proportion of total isolates with phenotypic susceptibility and MBT-S results in agreement (i.e., carbapenem susceptible/MBT-S CfiA negative or carbapenem non-susceptible/MBT-S CfiA positive). CA compared to ertapenem and meropenem susceptibility per CLSI was 90.3%, 95.1%, respectively, and 93.2%, 96.1% using EUCAST breakpoints. Regardless of carbapenem or interpretive criteria, VME and ME, defined as the proportion of carbapenem non-susceptible testing CfiA negative by MBT-S and the proportion of carbapenem susceptible isolates testing positive for CfiA by MBT-S, respectively, exceeded 3.0% (Table 3).

**TABLE 3:** Evaluation of Bruker MALDI Biotyer Subtyping module across IHMA and BJH B. fragilis *sensu stricto* isolates (*n* = 103)

	CLSI breakpoints		EUCAST breakpoints	
	Ertapenem	Meropenem	Ertapenem	Meropenem^*a*^
PPA (95% CI)	93.8 (67.7–99.7)	95.2 (74.1–99.8)	84 (63.1–94.7)	95.2 (74.1–99.8)
NPA (95% CI)	89.6 (80.1–94.9)	95.1 (87.3–98.4)	96.2 (88.4–99.0)	96.3 (88.8–99.0)
CA	90.3% (93/103)	95.1% (98/103)	93.2% (96/103)	96.1% (99/103)
VME	6.3% (1/16)	4.8% (1/21)	16.0% (4/25)	4.5% (1/22)
ME	10.3% (9/87)	4.9% (4/82)	3.8% (3/78)	3.7% (3/81)

Abbreviations: PPA, positive percent agreement; NPA, negative percent agreement; CI, confidence interval; CA, category agreement; VME, very major error rate; ME, major error rate.

a Total *n* = 102, excludes 1 IHMA isolate with meropenem MIC = 4 (EUCAST meropenem breakpoints: susceptible ≤2, resistant >8).

10.1128/mbio.03603-21.8 TABLE S1 Observed and calculated predictive values of Bruker MALDI Biotyer Subtyping module at various rates of carbapenem-resistant B. fragilis *sensu stricto*. Download Table S1, DOCX file, 0.01 MB. Copyright © 2022 Wallace et al. 2022 Wallace et al. https://creativecommons.org/licenses/by/4.0/ This content is distributed under the terms of the Creative Commons Attribution 4.0 International license.

Approximately half of the BFSS isolates from BJH (*n* = 38, 45%) and nearly all IHMA isolates (*n* = 19, 95%) were positive for *cfiA* by previously established PCR methods (Data Set S1, Table 2) (18). An amplicon of a size consistent with *cfiA* was also detected among 47% of non-*fragilis Bacteroides* species (*n* = 43) which represented the majority of *B. ovatus* (78%) and P. distasonis isolates. To our knowledge, this is the first report of *cfiA* detection in non-*fragilis*-BFG. However, the AMRfinder tool did not detect any genes annotated as *cfiA* within these strains. Of the *cfiA*-PCR positive BFSS isolates, 2.4% (2/83) from BJH and 47.4% (9/19) from IHMA had an amplicon consistent with an upstream IS, while none of the non-fragilis BFG had *cfiA*-associated IS elements detected by PCR. Among carbapenem-resistant BFSS, 52% (11/21) were positive for both *cfiA* and an upstream IS by PCR. All *IS* PCR positive isolates (*n* = 11) were confirmed by ISfinder or BLAST, with one additional isolate with a full-length *IS* sequence (IHMA_16) identified genomically. The forward primer binding sites (23) could not be found in the regions surrounding the IS element, which likely explains the negative PCR result. PCR-based methods for *cfiA*-associated IS detection appear accurate in comparison to other methods, while *cfiA* PCR frequently yielded several positive strains that were not validated by WGS or MBT-S (Data Set S1, Table 2).

Across the entire isolate cohort, IHMA_8 was the only carbapenem-resistant BFSS isolate with no detectable *cfiA* across all methods tested (Table 2). This isolate, IHMA_8, had MICs of 16 and >32 μg/mL to ertapenem and meropenem, respectively. Intriguingly, carbapenem resistance in this strain appears to be independent of IS activation of CfiA or the presence of the *cfiA* gene entirely. In addition to resolving *cfiA*-associated ISs, hybrid genome assembly allowed for complete resolution of a 14.6 kbp plasmid harboring the HMM-identified multispecies class A β-lactamase (*bla*) gene in IHMA_8 (Fig. S5A). The genomic architecture surrounding the *bla* gene was conserved among several isolates and had the structure of a mobilizable transposon (Fig. S5B) (24, 25). Unique to IHMA_8, however, an IS was apparent upstream of the *bla* gene, which could contribute to the elevated resistance to all β-lactams tested in this study (Fig. S5B).

10.1128/mbio.03603-21.5 FIG S5 Mobilizable elements carrying cryptic *bla* genes in BFGs. (A) A 14.6 kBp plasmid was identified in IHMA_8, which harbors the multispecies *bla* gene on a mobilizable transposon. Genes within the mobilizable transposon are purple while the remaining genes are cyan. GC skew was generated by DNAPlotter. (B) Easyfig contig alignments suggest multispecies *bla* gene is carried on a mobilizable transposon similar to previously reported elements in BFG species (24, 25). Download FIG S5, TIF file, 0.5 MB. Copyright © 2022 Wallace et al. 2022 Wallace et al. https://creativecommons.org/licenses/by/4.0/ This content is distributed under the terms of the Creative Commons Attribution 4.0 International license.

### Division I and II B. fragilis SS isolates fall into distinct genomospecies categorizations.

**FIG 5:**
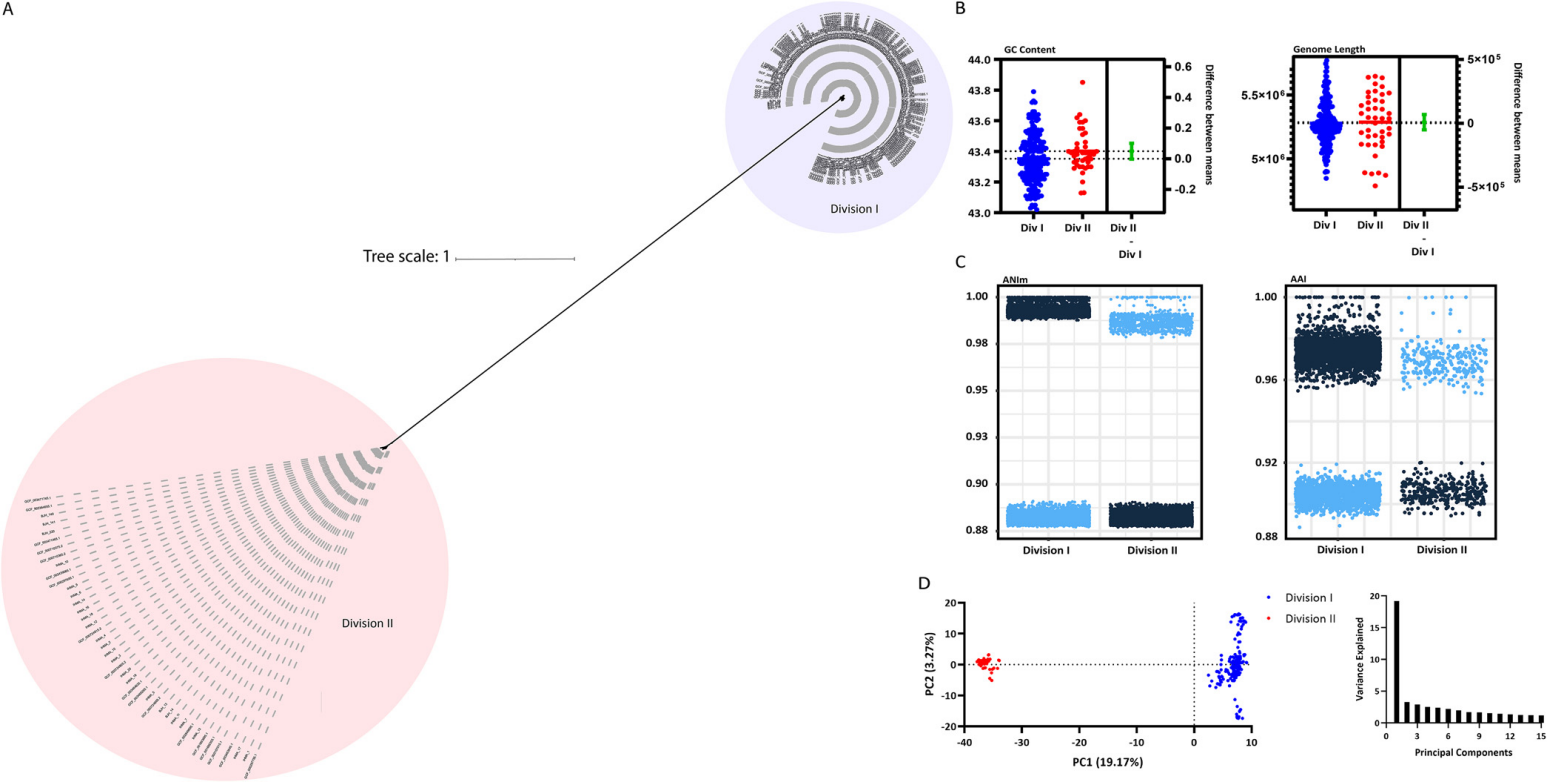
Comparative genomic analyses between division I and division II BFSS. (A) Unrooted, maximum likelihood phylogenetic tree generated from a core genome alignment of all BFSS strains in this study as well as available assemblies from NCBI. (B) GC content and genome lengths were compared between the two BFSS divisions. The difference between the means for both GC content and length was not statistically significant as determined by an unpaired two-tailed *t* test (Prism v9.0.0). (C) Pairwise average nucleotide identity (ANI) and average amino acid identity (AAI) of BFSS genomes. Data are stratified on the *x*-axis by division of the first isolate within the isolated pair and data points are colored by division status of the corresponding division of the partner isolate (dark blue = division I, light blue = division II) (D) Principal-component analysis (PCA) based on Jaccard distance matrix of the accessory genome of BFSS strains and scree plot of principal coordinate eigenvalues.

Previous studies have revealed that BFSS isolates segregate into two divisions which are distinguished by the mutually exclusive presence of endogenous *cepA* (division I) or *cfiA* (division II) (11). Our investigations further revealed that division II isolates appear to be distinct genomospecies due to phylogenetic clustering (Fig. 2A) and ANI values below the threshold for species-level identification with type BFSS strains (Fig. 2C). A genome alignment of the division I reference genome from NCTC 9343 and the division II isolate IHMA_4, which had a closed, circular genome through hybrid assembly, revealed that *cepA* and *cfiA* are present in distinct regions of the genome (Fig. S6). To further compare division I and division II strains, all available BFSS assemblies from NCBI as well as isolate assemblies from this study were used to generate a core genome alignment and an unrooted, maximum likelihood BFSS phylogeny tree (Fig. 5A). All division I and division II strains again formed two distinct clades. Type assemblies, most BJH assemblies, and the representative commensal genomes were in the division I clade, while most IHMA strains and the five *cfiA*-positive BJH strains were division II. Guanine-cytosine (GC) content, as well as genome length values generated from the assemblies, were not significantly different between the two divisions (Unpaired *t* test, GC content: *P* = 0.06, genome length: *P* = 0.78) (Fig. 5B). Closer inspection of pairwise ANI and AAI values of the complete BFSS genome collection revealed distinct clustering for both values that was stratified by division status, with division II ANI values well below the species cutoff of 95% relative to all division I strains (Fig. 5C) (12). Finally, a principal-component analysis revealed much of the variance within the accessory genome is due to division I or II status, as these two divisions clustered distinctly across the PC1 axis (PC1 = 19.17% variance explained) (Fig. 5D). Altogether, division II BFSS, which accounts for most of the resistance to the last resort agent carbapenem in BFG, is genetically distinct from division I and is, by accepted metrics (12), a distinct genomospecies.

10.1128/mbio.03603-21.6 FIG S6 Whole-genome alignment of division I BFSS strain NCTC 9343 and division II strain IHMA 4. Alignment was performed using Mauve v20150226. Loci containing the *cepA* and *cfiA/ccrA* genes specific to division I and division II BFSS, respectively, are shown. Download FIG S6, TIF file, 0.7 MB. Copyright © 2022 Wallace et al. 2022 Wallace et al. https://creativecommons.org/licenses/by/4.0/ This content is distributed under the terms of the Creative Commons Attribution 4.0 International license.

## DISCUSSION

Anaerobic bacteria can cause life-threatening infections and present unique challenges in terms of antimicrobial resistance, yet species-level identification and susceptibility testing are not routinely performed in many clinical labs. Previous studies demonstrate BFG species are the most frequent cause of anaerobic infection (2), yet comprehensive characterization from a phenotypic, diagnostic, and genomic perspective has not been recently reported on contemporary isolates. Within this study, we establish the crucial link between species and resistance profiles to frontline antibiotics. MALDI-ToF identification of BFG isolates to species-level were verified with a phylogenetic analysis of WGS assemblies. Although most species within BFG (B. fragilis, B. vulgatus, B. ovatus, B. thetaiotaomicron, B. caccae, and B. pyogenes) are claimed indications of both FDA-cleared MALDI-ToF platforms for microorganism identification, some laboratories report these organisms to the group level. Our findings indicate that MALDI-ToF-based species-level identifications are not only reliable but may also be clinically informative as distinct resistance profiles between members of the BFG were observed. Elevated MICs among non-fragilis *Bacteroides* spp. to piperacillin-tazobactam compared to mostly susceptible BFSS isolates was most profound (Fig. 3 and Table 1). These findings are consistent with previous reports of national anaerobic antimicrobial susceptibility surveys (6, 8). Furthermore, the frequency of each unique BFG species as well as the variety of infection sites in our local BJH collection is reflective of previous reports (2, 8, 9), strengthening the ability to translate these findings to cohorts in other locations.

Despite growing evidence of stark differences in resistance patterns against both β-lactam/β-lactamase inhibitor combination agents and carbapenems between BFSS and non-*fragilis Bacteroides* species and congruent enrichment of specific β-lactamases, the mechanistic basis of species-dependent resistance has not been entirely explained. This raises multiple questions worth further study. Alterations in the penicillin-binding protein (PBP) target or modifications that promote efflux or alter cell permeability to confer β-lactam resistance, for example, may be alternative mechanisms worth comprehensively exploring in BFG (26, 27). We determined there were at least eight distinct PBPs and 10 porins in the BFSS isolates in this study using a BLASTP search (Fig. S3B and C). Additional copies of PBP2xBfr and PBP4Bfr were noted in BJH_7 and BJH_108, respectively, while PBP2Bfr was missing in IHMA_15. IHMA_15, which was meropenem resistant and had reduced ertapenem susceptibility, was the only strain exhibiting loss of a porin coding region. Among division I strains, BJH_108 had an elevated piperacillin-tazobactam MIC (2 μg/mL, BFSS MIC_50_ = 1 μg/mL). The BLASTP identities for division II BFSS were found to vary substantially for most of the PBPs while identities below 90% were generally only observed for two porins in this division (Fig. S3D and E). Although PBP and porin populations of BFSS are moderately defined in the literature, further studies are needed to confirm the potential effects of specific resistance mutations or gene loss on β-lactam susceptibility.

A MALDI-ToF application validated in this study is the detection of CfiA for early warning resistance prediction in BFSS isolates by the Bruker Subtyper module (MBT-S). Because anaerobic susceptibility testing is not often performed in most clinical laboratories, clinical resistance is likely underreported. Herein we report that the MBT-S demonstrated 94 to 95% PPA and 90 to 95% NPA compared to carbapenem susceptibly interpreted by CLSI guidelines (Table 3). Among consecutively collected BFSS isolates from BJH only, this resulted in a PPV of 50% and NPV ranging from 96 to 100%. Category agreement compared to carbapenem susceptibly was also ≥ 90%. These data suggest that the Bruker MBT-S is most useful as a carbapenem resistance rule-out in BFSS isolates routinely in clinical labs. Importantly, presumptive CfiA detection by MBT-S did not always correspond to phenotypic carbapenem resistance as evidenced by VME and ME rates > 3%, unacceptable by the FDA for new antimicrobial susceptibility test systems. As such, MBT-S CfiA detection should be followed-up with additional testing to confirm carbapenemase-mediated resistance particularly in settings with carbapenem-resistant BFSS rates of < 1% where the MBT-S PPV is expected to be <50% (Table S1). Modifications of widely utilized rapid phenotypic carbapenemase assays, such as the mCIM or Carba-NP may be effective strategies to confirm MBT-S presumptive CfiA detections readily in the clinical lab. Nonetheless, MALDI-ToF MS appears to be a powerful tool that can yield important insights into the possible resistance of this diverse organism group.

Although most carbapenem resistance in BFSS is thought to be *cfiA*-mediated, *cfiA*-independent resistance has also been reported. In this cohort, IHMA_8 had high-level resistance to ertapenem (MIC = 16), meropenem (≥32), and piperacillin-tazobactam (≥256). However, PCR and WGS-based methods failed to detect a *cfiA* gene or IS element. IHMA_8 encoded *cepA* classifying it within division I BFSS isolates that are rarely carbapenem-resistant. Division I strains with elevated resistance to carbapenems and piperacillin-tazobactam often had other β-lactam ARG classes in addition to *cepA*, including IHMA_8 which had a mobilized class A *bla* gene identified by HMM profiling that was located downstream of an IS element (Fig. S5). Several *bla* genes were identified in our BFG isolate collection through HMM, but these genes do not appear to have been previously characterized or annotated in resistance gene databases. Additional studies characterizing the activity and spectrum of these putative β-lactamases in anaerobic bacteria are warranted.

In non-*fragilis Bacteroides* species, much less is known about the mechanism of carbapenem resistance. Detection of *cfiA* has not been described in these species but we found that nearly half of non-*fragilis Bacteroides* species in our isolate cohort had amplicons consistent with a *cfiA* PCR product (Data set S1). However, we were unable to detect genes annotated as *cfiA* in genomic assemblies or analysis of raw (preassembly) Illumina short-read sequencing reads in PCR positive non-*fragilis Bacteroides* isolates. While PCR artifact or contamination cannot be excluded, additional studies investigating the potential for distant *cfiA* homologs, interrupted pseudogenes, or *cfiA* regions that are not captured in the conventional short-read assembly process are needed to verify this finding.

Interestingly, we found that phenotypic resistance profiles were not always interpreted consistently when different breakpoints (CLSI or EUCAST) were applied. For example, across all species, decreased susceptibility to piperacillin-tazobactam and carbapenems was observed when using EUCAST versus CLSI interpretive criteria due to a lower susceptible breakpoint for ertapenem (≤0.5 μg/mL per EUCAST versus ≤4 μg/mL per CLSI), meropenem (≤2 μg/mL per EUCAST versus ≤4 μg/mL per CLSI), and piperacillin-tazobactam (≤8 per EUCAST versus ≤16 per CLSI) (Table 1). Additional studies evaluating clinical outcomes and MIC distributions of various species within the BFG are needed to determine whether species-level reporting and clinical breakpoints are warranted.

Among BFSS isolates, we uncovered a striking genetic divergence between division I and division II species, which is for the first time described through comparative genomics methods in this study. The CfiA carbapenemase is only present in division II strains, yet all reported type assemblies and many commensal BFSS genomes (16) are division I strains. The source of anaerobic infection is often commensal microbes (2), which begs the question of whether division II BFSS can exist as a commensal, serving as a reservoir for highly resistant infections.

We note important limitations to our study. Most isolates studied (174/194) were from a single medical center, so the translation of these results where BFG resistance rates vary by geographic region is unclear. Furthermore, information on clinical treatment outcomes and the impact of rapid presumptive *cfiA* detection were not evaluated. Nevertheless, we report the largest comparative genomic analysis of a BFG cohort to date, which can serve as a basis for future comparisons to data sets from other regions. Paired with robust clinical data sets on the source, diagnosis, and phenotypic resistance profiles, these findings inform our understanding and management of BFG infection.

In summary, we present a comprehensive study of 194 BFG isolates, which are a dominant cause of anaerobic infection. We leveraged comparative genomics to confirm the accuracy of two widely used MALDI-ToF MS technologies in resolving species-level identities and cfiA prediction, the importance of which is emphasized by our findings of striking species-dependent phenotypic and ARG profiles to commonly administered antibiotic agents. We report *cfiA/IS*-dependent and independent mechanism of activating resistance to carbapenems in this cohort and validate multiple tools for *cfiA* and IS detection. Finally, our data assert division II BFSS, which is identified at high frequency in carbapenem-resistant BFG infections, as a distinct genomospecies from division I BFSS.

## MATERIALS AND METHODS

### Clinical study isolates and MALDI-ToF MS analysis.

A total of 174 Bacteroides fragilis group (BFG) consecutively available isolates recovered from clinical specimens submitted to the Barnes Jewish Hospital clinical microbiology laboratory (St. Louis, MO) from February 2018 to July 2018 were evaluated in this study. To increase the number of carbapenem-resistant BFG evaluated, 20 BFSS isolates resistant to a carbapenem class agent were acquired from the International Health Management Associates (IHMA) bacterial repository for evaluation, and *cfiA*-positive BFSS isolates obtained from the Barnes-Jewish clinical lab collected through 2020 were also included. Isolates were subcultured on prereduced anaerobically sterile (PRAS) Brucella (Hardy Diagnostics) and incubated in anaerobic conditions for species-level identification with 2 commercial matrix-assisted laser desorption ionization-time of flight mass spectrometry (MALDI-ToF MS) systems: MALDI Biotyper (Bruker Daltonics) Research Use Only (RUO) Database BDALv7 and VITEK-MS (bioMérieux) RUO Knowledge Base v. KB 3.0 per manufacturer’s instructions. For *cfiA* detection, the Bruker MBT Subtyper module was enabled and simultaneously used during spectra acquisition for isolate identification. Isolates identified as B. fragilis *sensu stricto* (BFSS) with a high confidence score (>2.0) received a presumptive *cfiA* positive or negative determination.

### Anaerobic antimicrobial susceptibility testing.

Clinical BFG isolates were evaluated by gradient diffusion method for susceptibility to antimicrobial agents piperacillin-tazobactam (LiofilChem), ertapenem, meropenem, and metronidazole (bioMérieux). BFG isolates cultured on PRAS Brucella blood agar for 24 to 28 h were used to make 1 McFarland standard organism suspension in Brucella broth (BD BBL™) which was then lawn-struck onto PRAS Brucella blood agar plates. Gradient diffusion strips were placed on inoculated plates and incubated at 35°C in EZ Anaerobe Gas-pack systems (BD) for up to 72 h. MICs were read at 72h. Species-specific differences in MIC were investigated using Tukey’s multiple-comparison test with ordinary one-way ANOVA (GraphPad Prism v9.0.0) to detect significant differences between the geometric mean of each species group (defined as an adjusted *P* value <0.05).

### Genomic DNA extraction and molecular detection of carbapenemase genetic determinants cfiA and IS.

BFG isolates stored at −80°C were cultured onto Brucella blood agar and grown in anaerobic conditions for 48 h at 35°C. Fresh (<72 h) BFG isolates from Brucella blood agar incubated anaerobically were harvested and resuspended in nuclease-free, molecular grade water and frozen at −20°C until DNA extraction. Genomic DNA was extracted from each isolate using the QIAamp/MoBio BiOstic Bacteremia DNA kit (Qiagen).

End-point PCR was used to detect *cfiA* and upstream insertions sequence elements (*IS*) from all BFG isolates using previously described methods (23). Briefly, illustra™ puRe *Taq* Ready-to-Go PCR beads (GE Healthcare, UK) were suspended with a master mix containing 100 ng of gDNA, forward (GBI-1, 5′-CCCAACTCTCGGACAAAGTG) and reverse (GBI-2, 5′-AGTGAATCGGTGAATCCATG) primers at a final concentration of 0.2 μM and sterile nuclease-free water up to a final volume of 25 μL. For *cfiA* PCR the reaction was performed under the following conditions: 95°C for 5 min for initial denaturation, then 35 cycles of 95°C for 20 sec, 64°C for 2 min, 72°C for 30 sec, followed by a 5 min final extension at 74°C. For *IS* PCR, Ready-to-Go PCR beads were suspended with a master mix as described above with *IS* forward (G, 5′-CGCCAAGCTTTGCCTGCCATTAT) and reverse (E, 5′-CTTCGAATTCGGCGAGGGATACATAA) primers (18). Following an initial denaturation of 95°C for 5 min, the reaction was cycled 35 times at 95°C for 20 sec, 64°C for 2 min, 74°C for 3 min, followed by a final extension of 74°C for 5 min. PCR amplicons were visualized on a 2% agarose gel. Expected amplicon sizes were 350 bp for *cfiA* PCR and1.6 to 1.7Kb for IS PCR. Each PCR batch was run with positive (*cfiA*+/IS+: WIS-ImiR-001, *cfiA*+/IS-: B. fragilis ATCC 25285™), negative (*cfiA*-/IS-: B. thetaiotaomicron ATCC 29741™) and no template controls that performed as expected on all valid runs.

### Whole-genome sequencing and *de novo* genome assembly.

For whole-genome sequencing, 0.5 ng gDNA for each isolate was used to prepare Illumina sequencing libraries using the Nextera kit (Illumina, San Diego, CA, USA) (28). All libraries were pooled at equal concentrations and sequenced at a target depth of 2 million reads per sample (2 × 150 bp) on the NextSeq 500 High Output platform (Illumina, San Diego, CA, USA).

Adapters were removed from the demultiplexed reads via Trimmomatic (v0.38) (29), and read qualities were assessed using FastQC (v0.11.7) (29). Genome assemblies were built using Unicycler (v0.4.7) (30), and assembly quality was evaluated using Quast (v4.5). *De novo* assemblies were sequenced at ≥40× sequencing depth with a total number of contigs ≤300 and N50 ≥10,000 were selected for further analyses. In some cases, additional reads were obtained to achieve at least 40× coverage. Completeness and contamination were evaluated using CheckM (v1.0.13) (31) to ensure completeness values ≥95% and contamination ≤5%. Prokka (v1.14.5, default parameters, contigs >500 bp) (19) was used to annotate high-quality genomes.

### Hybrid genome assembly.

Select strains underwent Oxford Nanopore long-read sequencing and hybrid assembly (BJH_13, BJH_14, BJH_140, BJH_141, IHMA_1, IHMA_2, IHMA_3, IHMA_4, IHMA_6, IHMA_8, IHMA_9, IHMA_10, IHMA_13, IHMA_16, IHMA_17, IHMA_18, IHMA_19, IHMA_20). BFG cells were cultivated and harvested as described previously and stored at −80°C. High molecular weight DNA was extracted using the phenol-chloroform extraction method, and the gDNA was stored at 4°C for up to 1 week. Libraries were prepared using the Oxford Nanopore Ligation Sequencing and Native Barcode kits. Filtlong was used to subsample the long reads to 100× sequencing depth. Unicycler (30) was employed for hybrid assemblies of each genome using both long reads and Illumina short reads.

As required for BJH_14, manual finishing of the IS-cfiA region was carried out using minimap2 (v2.14) and samtools (v1.9). Long reads with a minimum length of 2000 nucleotides that aligned with the putative IS-cfiA query sequence with 85% identity over a minimum of 1000 nucleotides were extracted and aligned with the query sequence. This resulted in 185 reads extracted and aligned with a mean depth of coverage of 144× over the IS-cfiA query sequence (samtools v1.12).

### Comparative analyses.

Roary v3.12.0 (32) was used for core genome alignments of *de novo* short-read assemblies and deposited assemblies, with a BLASTP cutoff of 70 for alignment of the entire BJH isolate collection with Type assemblies and a BLASTP cutoff of 90 for the B. fragilis SS alignment. Neighbor-joining phylogenetic trees were constructed from these alignments using FastTree v2.1.7. ANI values were determined using pyANI (https://github.com/widdowquinn/pyani). The maximum-likelihood tree for Fig. 5 was constructed from the B. fragilis SS alignment using raxml v8.2.11 (GTRGAMMA model, 1,000 bootstraps). GC content and genome lengths were compared using an unpaired two-tailed *t* test (Prism v9.0.0). PCA of accessory genomes was performed using the Vegan package of R functions (33) with accessory genes identified from the Roary output for the B. fragilis SS core genome alignment as input.

### ARG and associated insertion sequence identification.

AMRfinder (v3.8.4) was employed to detect ARGs. For the detection of PBPs and porins, protein BLAST was carried out using BLAST+ (v2.9.0) using an E value cutoff of 1×10^−20^ and a percentage identity cutoff of 50%. Insertion sequences were identified upstream of the *cfiA* gene in Division II BFSS genomes using the built-in ISFinder function of Prokka as well as the ISFinder BLAST tool to generate additional IS sequence characterization for Table 2 (20).

### Data Availability.

Raw sequencing data and whole-genome assemblies are available under BioProject ID PRJNA745162.

## References

[1] Brook I. 2016. Spectrum and treatment of anaerobic infections. J Infect Chemother 22:1–13. doi:10.1016/j.jiac.2015.10.010. 26620376

[2] Wexler HM. 2007. Bacteroides: the good, the bad, and the nitty-gritty. Clin Microbiol Rev 20:593–621. doi:10.1128/CMR.00008-07. 17934076 PMC2176045

[3] Wexler AG, Goodman AL. 2017. An insider's perspective: bacteroides as a window into the microbiome. Nat Microbiol 2:17026. doi:10.1038/nmicrobiol.2017.26. 28440278 PMC5679392

[4] Lassmann B, Gustafson DR, Wood CM, Rosenblatt JE. 2007. Reemergence of anaerobic bacteremia. Clin Infect Dis 44:895–900. doi:10.1086/512197. 17342637

[5] Kim J, Lee Y, Park Y, Kim M, Choi JY, Yong D, Jeong SH, Lee K. 2016. Anaerobic Bacteremia: impact of Inappropriate Therapy on Mortality. Infect Chemother 48:91–98. doi:10.3947/ic.2016.48.2.91. 27433379 PMC4945732

[6] Snydman DR, Jacobus NV, McDermott LA, Golan Y, Hecht DW, Goldstein EJ, Harrell L, Jenkins S, Newton D, Pierson C, Rihs JD, Yu VL, Venezia R, Finegold SM, Rosenblatt JE, Gorbach SL. 2010. Lessons learned from the anaerobe survey: historical perspective and review of the most recent data (2005–2007). Clin Infect Dis 50 Suppl 1:S26–33. doi:10.1086/647940. 20067390

[7] Snydman DR, Jacobus NV, McDermott LA, Golan Y, Goldstein EJ, Harrell L, Jenkins S, Newton D, Pierson C, Rosenblatt J, Venezia R, Gorbach SL, Queenan AM, Hecht DW. 2011. Update on resistance of Bacteroides fragilis group and related species with special attention to carbapenems 2006–2009. Anaerobe 17:147–151. doi:10.1016/j.anaerobe.2011.05.014. 21664469

[8] Snydman DR, Jacobus NV, McDermott LA, Goldstein EJ, Harrell L, Jenkins SG, Newton D, Patel R, Hecht DW. 2017. Trends in antimicrobial resistance among Bacteroides species and Parabacteroides species in the United States from 2010–2012 with comparison to 2008–2009. Anaerobe 43:21–26. doi:10.1016/j.anaerobe.2016.11.003. 27867083

[9] Nagy E, Urban E, Nord CE, Bacteria ESGoARiA. 2011. Antimicrobial susceptibility of Bacteroides fragilis group isolates in Europe: 20 years of experience. Clin Microbiol Infect 17:371–379. doi:10.1111/j.1469-0691.2010.03256.x. 20456453

[10] Ferlov-Schwensen SA, Sydenham TV, Hansen KCM, Hoegh SV, Justesen US. 2017. Prevalence of antimicrobial resistance and the cfiA resistance gene in Danish Bacteroides fragilis group isolates since 1973. Int J Antimicrob Agents 50:552–556. doi:10.1016/j.ijantimicag.2017.05.007. 28666749

[11] Nagy E, Becker S, Soki J, Urban E, Kostrzewa M. 2011. Differentiation of division I (cfiA-negative) and division II (cfiA-positive) Bacteroides fragilis strains by matrix-assisted laser desorption/ionization time-of-flight mass spectrometry. J Med Microbiol 60:1584–1590. doi:10.1099/jmm.0.031336-0. 21680764

[12] Richter M, Rossello-Mora R. 2009. Shifting the genomic gold standard for the prokaryotic species definition. Proc Natl Acad Sci USA 106:19126–19131. doi:10.1073/pnas.0906412106. 19855009 PMC2776425

[13] Konstantinidis KT, Tiedje JM. 2005. Genomic insights that advance the species definition for prokaryotes. Proc Natl Acad Sci USA 102:2567–2572. doi:10.1073/pnas.0409727102. 15701695 PMC549018

[14] Qin QL, Xie BB, Zhang XY, Chen XL, Zhou BC, Zhou J, Oren A, Zhang YZ. 2014. A proposed genus boundary for the prokaryotes based on genomic insights. J Bacteriol 196:2210–2215. doi:10.1128/JB.01688-14. 24706738 PMC4054180

[15] Feldgarden M, Brover V, Haft DH, Prasad AB, Slotta DJ, Tolstoy I, Tyson GH, Zhao S, Hsu CH, McDermott PF, Tadesse DA, Morales C, Simmons M, Tillman G, Wasilenko J, Folster JP, Klimke W. 2020. Erratum for Feldgarden et al., “Validating the AMRFinder Tool and resistance gene database by using antimicrobial resistance genotype-phenotype correlations in a collection of isolates”. Antimicrob Agents Chemother 64:e00361-20. doi:10.1128/AAC.00361-20. 32209564 PMC7179288

[16] Zhao S, Lieberman TD, Poyet M, Kauffman KM, Gibbons SM, Groussin M, Xavier RJ, Alm EJ. 2019. Adaptive evolution within gut microbiomes of healthy people. Cell Host Microbe 25:656–667.e8. doi:10.1016/j.chom.2019.03.007. 31028005 PMC6749991

[17] Sydenham TV, Overballe-Petersen S, Hasman H, Wexler H, Kemp M, Justesen US. 2019. Complete hybrid genome assembly of clinical multidrug-resistant Bacteroides fragilis isolates enables comprehensive identification of antimicrobial-resistance genes and plasmids. Microb Genom 5:e000312. doi:10.1099/mgen.0.000312. PMC6927303 31697231

[18] Soki J, Fodor E, Hecht DW, Edwards R, Rotimi VO, Kerekes I, Urban E, Nagy E. 2004. Molecular characterization of imipenem-resistant, cfiA-positive Bacteroides fragilis isolates from the USA, Hungary and Kuwait. J Med Microbiol 53:413–419. doi:10.1099/jmm.0.05452-0. 15096551

[19] Seemann T. 2014. Prokka: rapid prokaryotic genome annotation. Bioinformatics 30:2068–2069. doi:10.1093/bioinformatics/btu153. 24642063

[20] Siguier P, Perochon J, Lestrade L, Mahillon J, Chandler M. 2006. ISfinder: the reference centre for bacterial insertion sequences. Nucleic Acids Res 34:D32–6. doi:10.1093/nar/gkj014. 16381877 PMC1347377

[21] Mahillon J, Chandler M. 1998. Insertion sequences. Microbiol Mol Biol Rev 62:725–774. doi:10.1128/MMBR.62.3.725-774.1998. 9729608 PMC98933

[22] Sakamoto M, Lan PTN, Benno Y. 2007. Barnesiella viscericola gen. nov., sp. nov., a novel member of the family Porphyromonadaceae isolated from chicken caecum. Int J Syst Evol Microbiol 57:342–346. doi:10.1099/ijs.0.64709-0. 17267976

[23] Kato N, Yamazoe K, Han CG, Ohtsubo E. 2003. New insertion sequence elements in the upstream region of cfiA in imipenem-resistant Bacteroides fragilis strains. Antimicrob Agents Chemother 47:979–985. doi:10.1128/AAC.47.3.979-985.2003. 12604530 PMC149317

[24] Tribble GD, Parker AC, Smith CJ. 1999. Genetic structure and transcriptional analysis of a mobilizable, antibiotic resistance transposon from Bacteroides. Plasmid 42:1–12. doi:10.1006/plas.1999.1401. 10413660

[25] Tribble GD, Parker AC, Smith CJ. 1997. The Bacteroides mobilizable transposon Tn4555 integrates by a site-specific recombination mechanism similar to that of the gram-positive bacterial element Tn916. J Bacteriol 179:2731–2739. doi:10.1128/jb.179.8.2731-2739.1997. 9098073 PMC179024

[26] Ayala J, Quesada A, Vadillo S, Criado J, Piriz S. 2005. Penicillin-binding proteins of Bacteroides fragilis and their role in the resistance to imipenem of clinical isolates. J Med Microbiol 54:1055–1064. doi:10.1099/jmm.0.45930-0. 16192437

[27] Dubreuil L, Odou MF. 2010. Anaerobic bacteria and antibiotics: what kind of unexpected resistance could I find in my laboratory tomorrow? Anaerobe 16:555–559. doi:10.1016/j.anaerobe.2010.10.002. 20971200

[28] Baym M, Kryazhimskiy S, Lieberman TD, Chung H, Desai MM, Kishony R. 2015. Inexpensive multiplexed library preparation for megabase-sized genomes. PLoS One 10:e0128036. doi:10.1371/journal.pone.0128036. 26000737 PMC4441430

[29] Bolger AM, Lohse M, Usadel B. 2014. Trimmomatic: a flexible trimmer for Illumina sequence data. Bioinformatics 30:2114–2120. doi:10.1093/bioinformatics/btu170. 24695404 PMC4103590

[30] Wick RR, Judd LM, Gorrie CL, Holt KE. 2017. Unicycler: resolving bacterial genome assemblies from short and long sequencing reads. PLoS Comput Biol 13:e1005595. doi:10.1371/journal.pcbi.1005595. 28594827 PMC5481147

[31] Parks DH, Imelfort M, Skennerton CT, Hugenholtz P, Tyson GW. 2015. CheckM: assessing the quality of microbial genomes recovered from isolates, single cells, and metagenomes. Genome Res 25:1043–1055. doi:10.1101/gr.186072.114. 25977477 PMC4484387

[32] Page AJ, Cummins CA, Hunt M, Wong VK, Reuter S, Holden MT, Fookes M, Falush D, Keane JA, Parkhill J. 2015. Roary: rapid large-scale prokaryote pan genome analysis. Bioinformatics 31:3691–3693. doi:10.1093/bioinformatics/btv421. 26198102 PMC4817141

[33] Dixon P. 2003. VEGAN, a package of R functions for community ecology. J Vegetation Science 14:927–930. doi:10.1111/j.1654-1103.2003.tb02228.x.

[34] Clinical and Laboratory Standards Institute. 2019. Performance Standards for Antimicrobial Susceptibility Testing, 29th ed CLSI Supplement M100, Wayne, PA.

[35] European Committee on Antimicrobial Susceptibility Testing. 2019. Breakpoint tables for interpretation of MICs and zone diameters. Version 9.0.

[36] Wexler HM, Read EK, Tomzynski TJ. 2002. Characterization of omp200, a porin gene complex from Bacteroides fragilis: omp121 and omp71, gene sequence, deduced amino acid sequences and predictions of porin structure. Gene 283:95–105. doi:10.1016/S0378-1119(01)00835-6. 11867216

[37] Wexler HM, Tenorio E, Pumbwe L. 2009. Characteristics of Bacteroides fragilis lacking the major outer membrane protein, OmpA. Microbiol 155:2694–2706. doi:10.1099/mic.0.025858-0. 19497947

[38] Philippon A, Slama P, Dény P, Labia R. 2016. A structure-based classification of class A β-lactamases, a broadly diverse family of enzymes. Clin Microbiol Rev 29:29–57. doi:10.1128/CMR.00019-15. 26511485 PMC4771212

