# Supplementary figures and images for: Comparative Genomics of Bacteroides fragilis Group Isolates Reveals Species-Dependent Resistance Mechanisms and Validates Clinical Tools for Resistance Prediction
Source: mBio. 2022 Jan 18;13(1):e03603-21. doi: 10.1128/mbio.03603-21 (PMC8764542; doi:10.1128/mbio.03603-21)

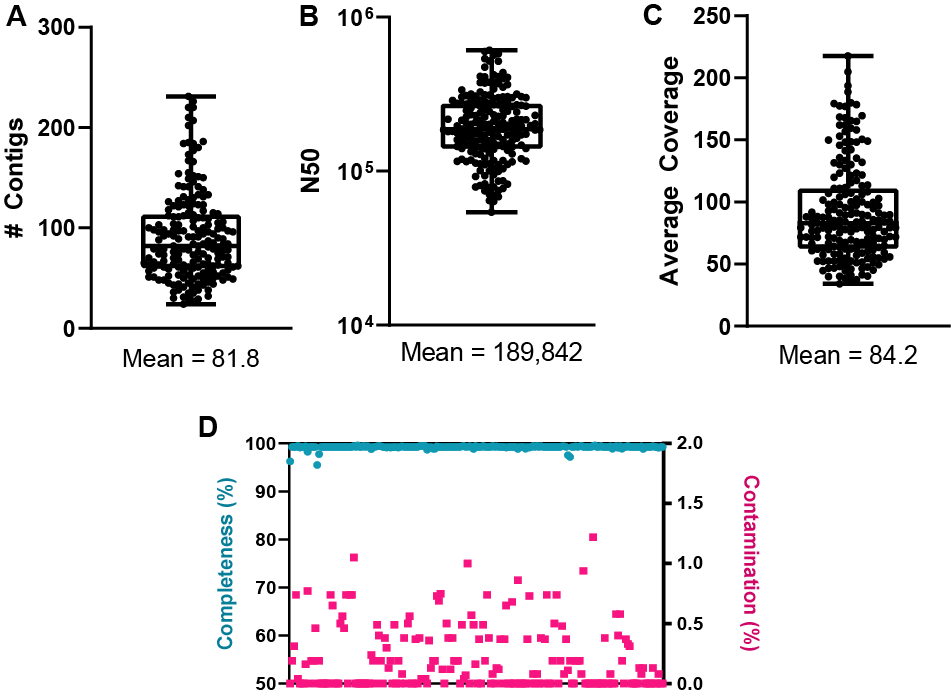

Supplement: FIG S1 [file mbio.03603-21-sf001.tif]

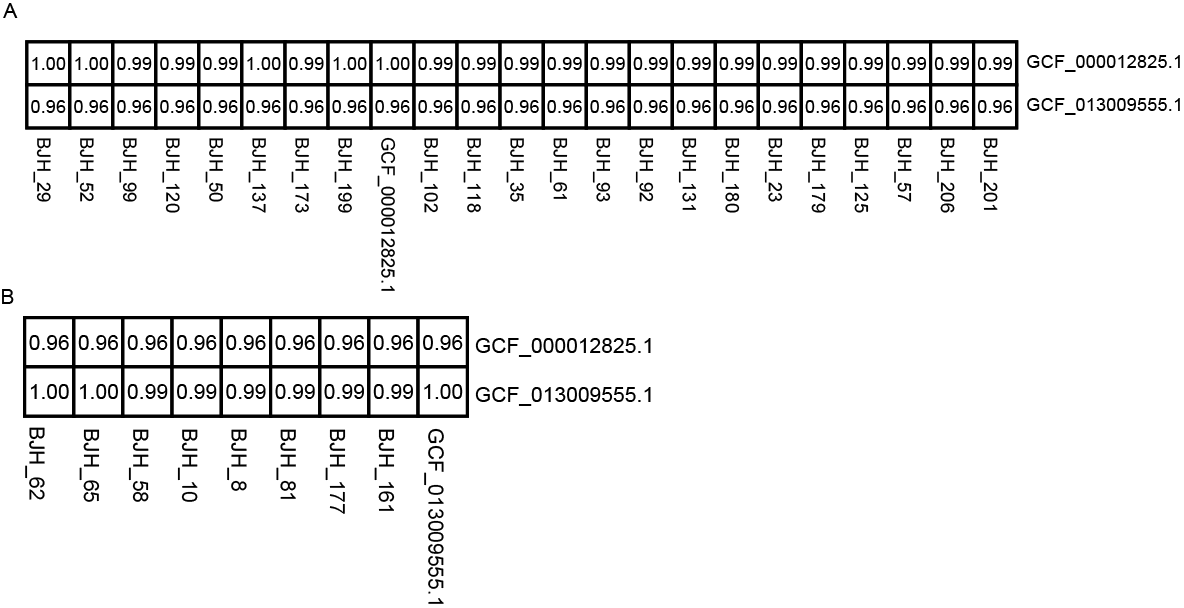

Supplement: FIG S2 [file mbio.03603-21-sf002.tif]

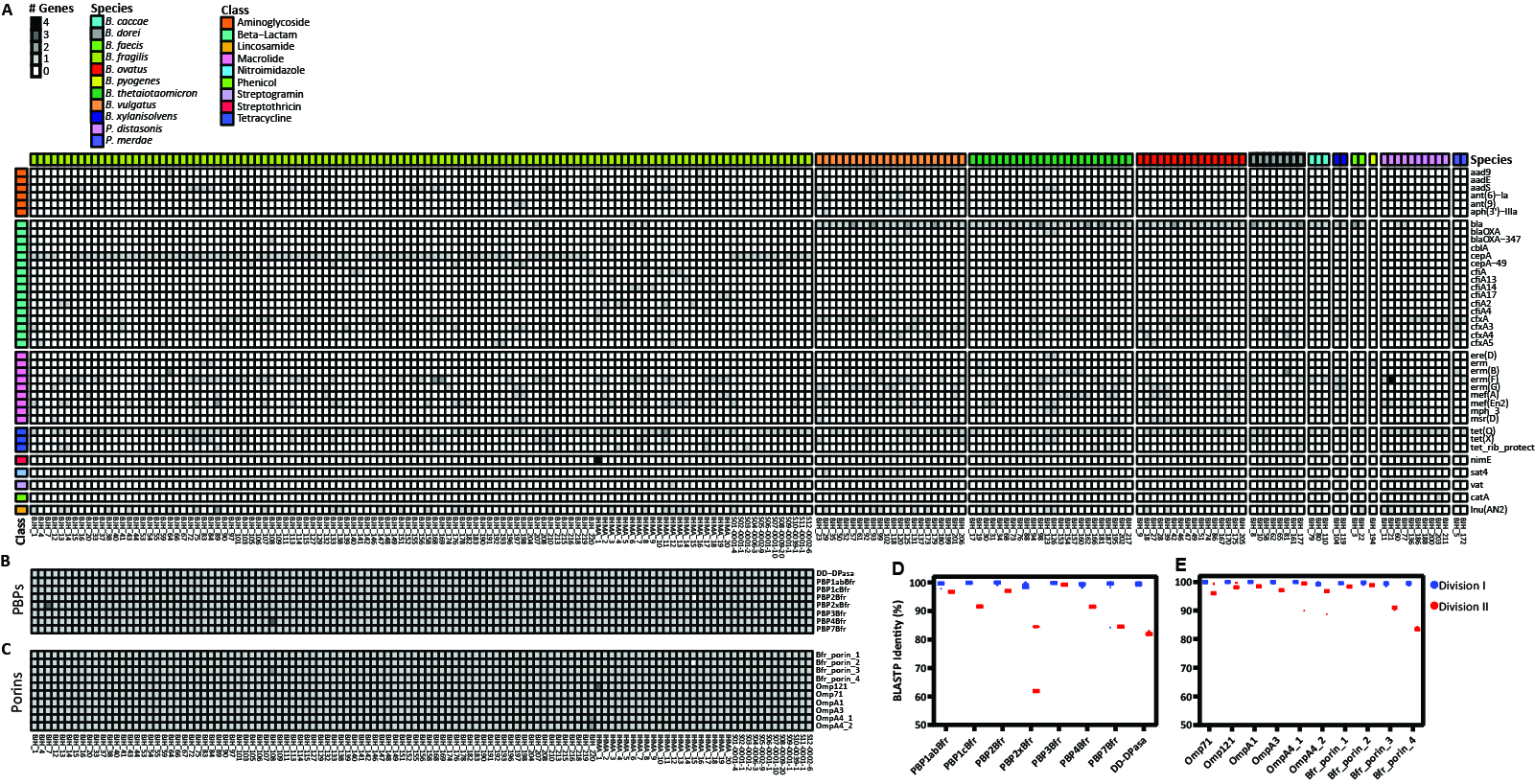

Supplement: FIG S3 [file mbio.03603-21-sf003.tif]

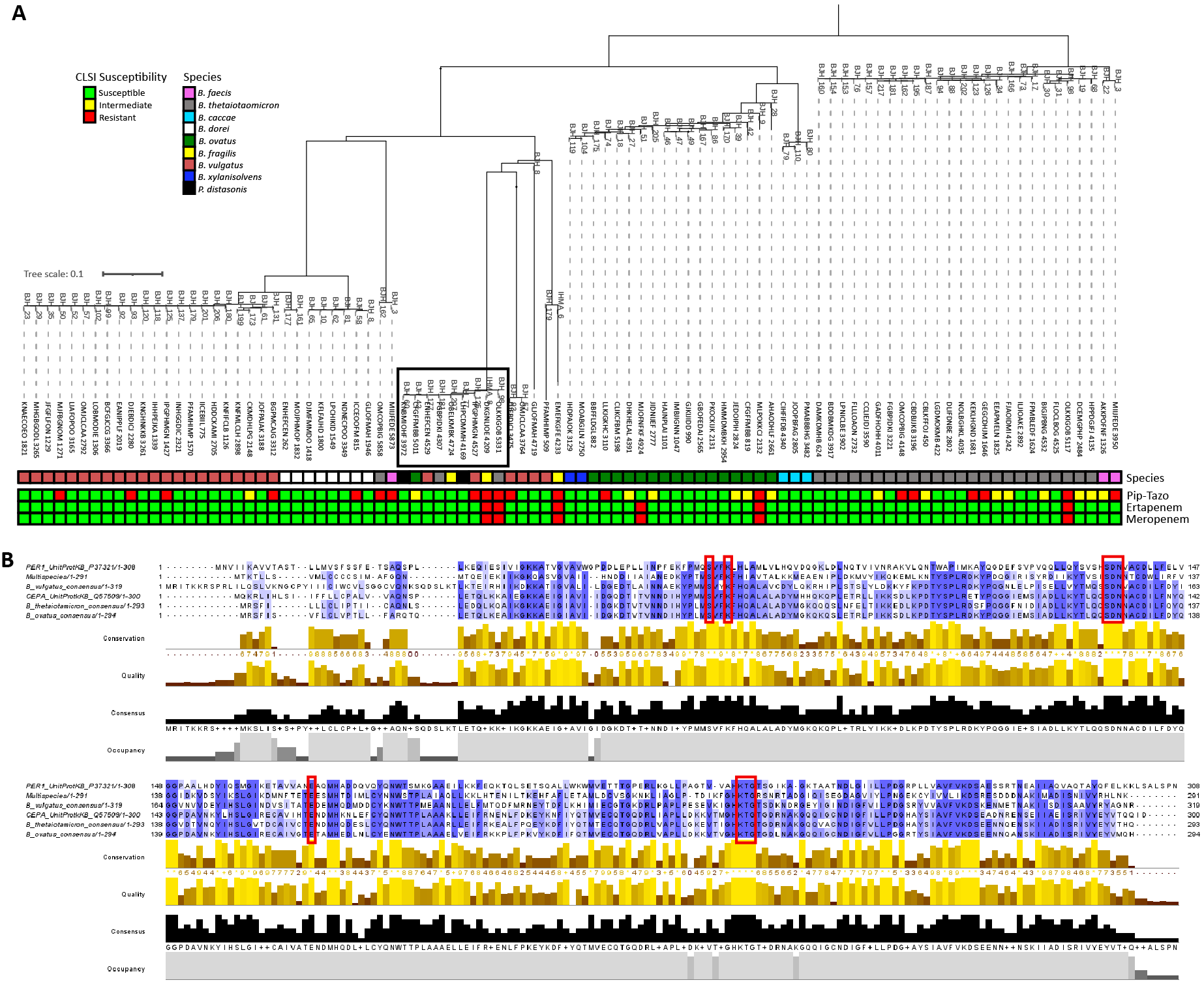

Supplement: FIG S4 [file mbio.03603-21-sf004.tif]

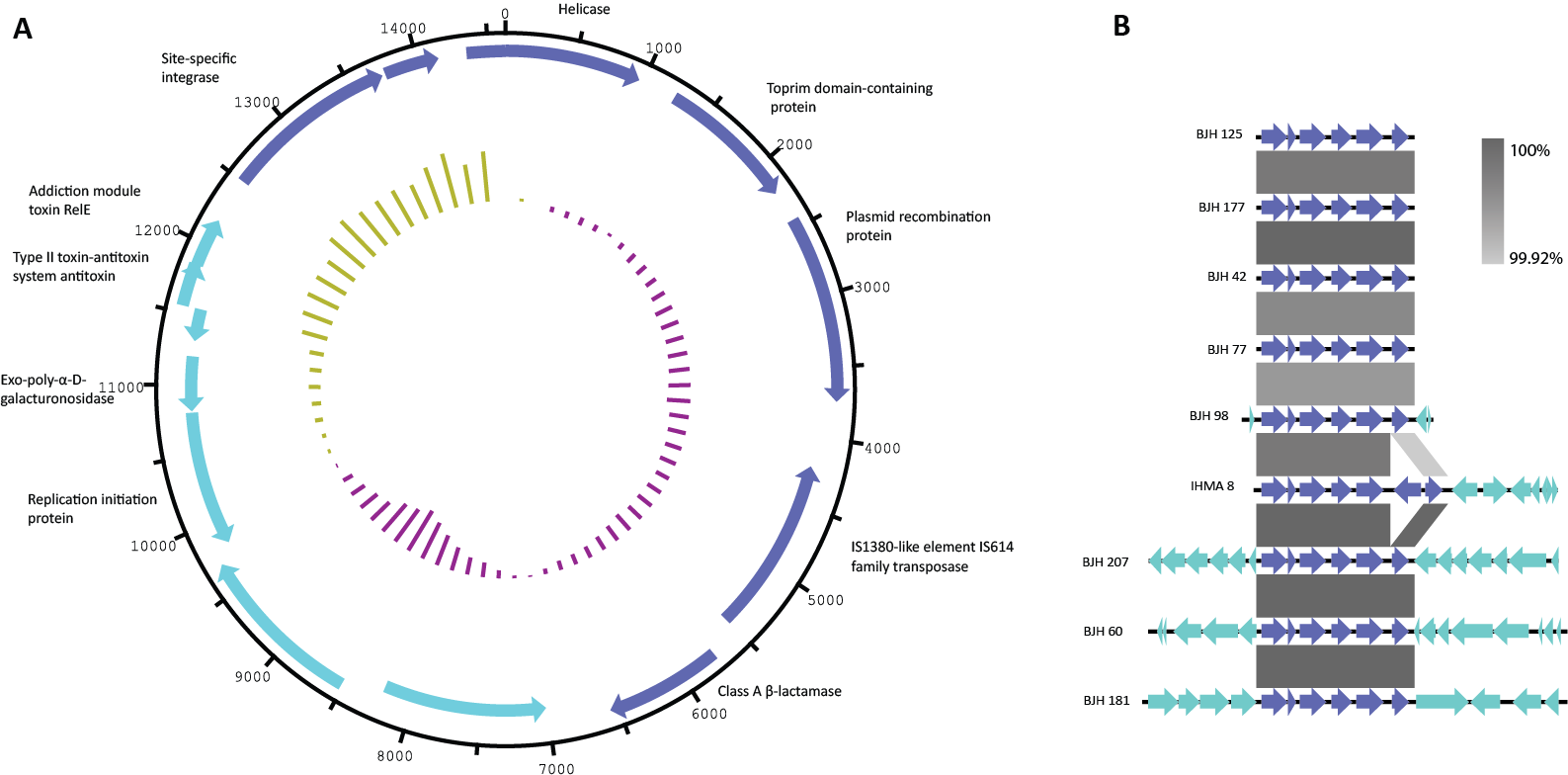

Supplement: FIG S5 [file mbio.03603-21-sf005.tif]

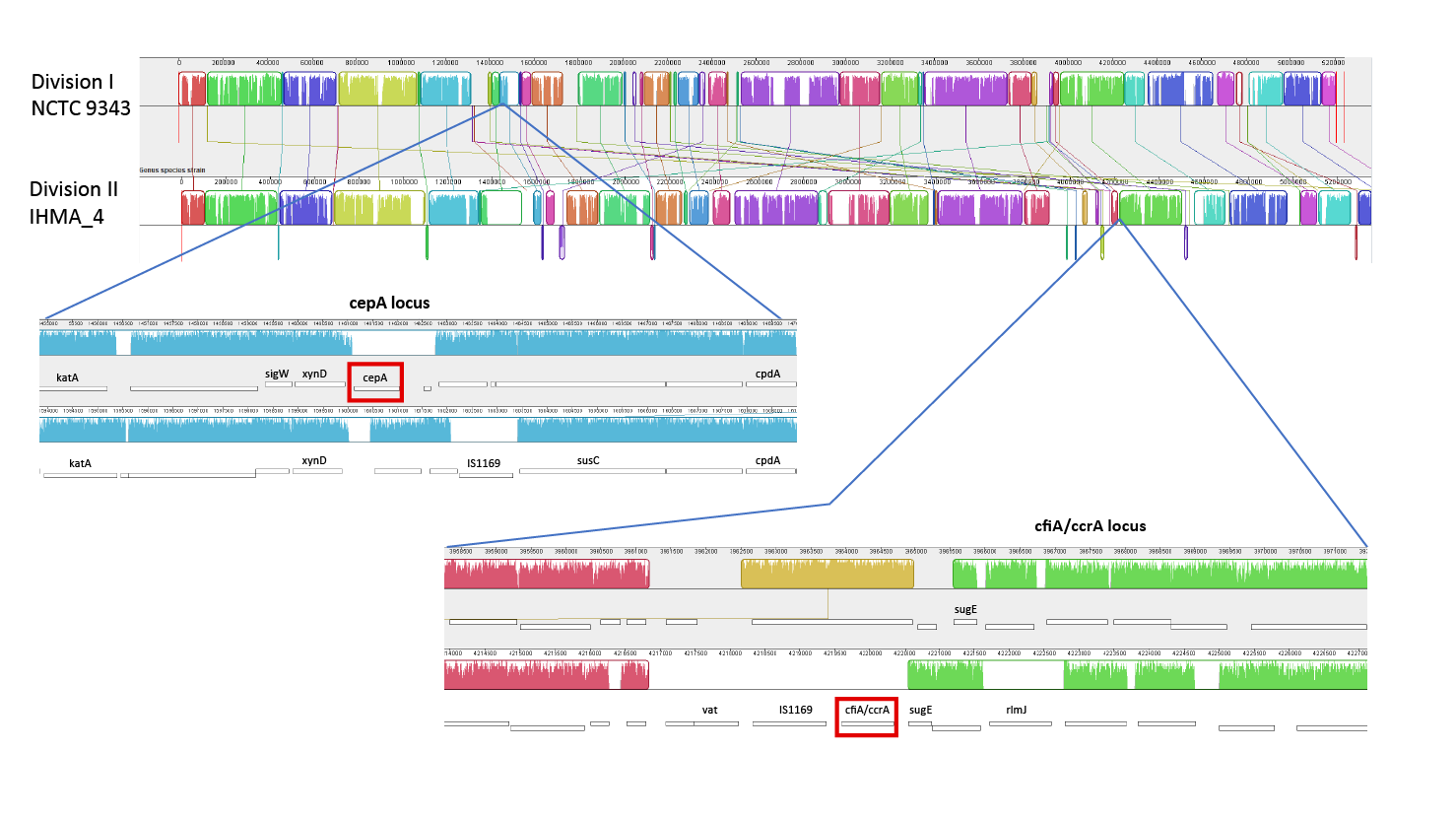

Supplement: FIG S6 [file mbio.03603-21-sf006.tif]
