# Supplementary material for: Comparative Genomics of Bacteroides fragilis Group Isolates Reveals Species-Dependent Resistance Mechanisms and Validates Clinical Tools for Resistance Prediction
Source: mBio. 2022 Jan 18;13(1):e03603-21. doi: 10.1128/mbio.03603-21 (PMC8764542; doi:10.1128/mbio.03603-21)
Supplement: TABLE S1 [file mbio.03603-21-st001.docx]

**Supplementary Table 1. Observed and calculated predictive values of Bruker MALDI Biotyer Subtyping module at various rates of carbapenem-resistant *B. fragilis sensu stricto*.**

|  | CLSI breakpoints | | EUCAST breakpoints | |
| --- | --- | --- | --- | --- |
|  | Ertapenem | Meropenem | Ertapenem | Meropenem |
| **2.4% prevalence**  **(Observed^1^)** |  |  |  |  |
| PPV (95% CI) | 50 (9.2-90.8) | 50 (9.2-90.8) | 50 (9.2-90.8) | 50 (9.2-90.8) |
| NPV (95% CI) | 100 (94.1-100) | 100 (94.1-100) | 96.1 (88.4-99) | 100 (94.1-100) |
| **0.25% prevalence** |  |  |  |  |
| PPV | 11.1% | 11.1% | 3.2% | 11.1% |
| NPV | 100% | 100% | 99.8% | 100% |
| **25% prevalence** |  |  |  |  |
| PPV | 94.3% | 94.3% | 81.6% | 94.3% |
| NPV | 100% | 100% | 82.9% | 100% |

^1^Observed prevalence in consecutively collected BJH *B. fragilis sensu stricto* isolates (n=82).
